# Chinese Herbal Formula, Huayu Tongbi Fang, Attenuates Inflammatory Proliferation of Rat Synoviocytes Induced by IL-1*β* by Regulating Proliferation and Differentiation of T Lymphocytes

**DOI:** 10.1155/2020/1706837

**Published:** 2020-05-19

**Authors:** Hening Chen, Changzhi Wang, Jinyu Li, Meiyier Huandike, Juan Liu, Qingchun Huang, Xue Li, Huijie Zhang, Jingwei Zhou, Limin Chai

**Affiliations:** ^1^Key Laboratory of Chinese Internal Medicine of Ministry of Education and Beijing, Dongzhimen Hospital, Beijing University of Chinese Medicine, Beijing 100700, China; ^2^Department of Rheumatology, The Second Affiliated Hospital of Guangzhou University of Chinese Medicine, Guangzhou 510000, China; ^3^Department of Rheumatology, Dongzhimen Hospital, Beijing University of Chinese Medicine, Beijing 100700, China

## Abstract

The inflammatory proliferation of fibroblast-like synoviocytes (FLSs) and functional imbalances in T lymphocytes play critical roles in the pathogenesis of rheumatoid arthritis (RA). The clinical efficacy of Huayu Tongbi Fang (HYTB, a traditional herbal formula) in RA treatment has been validated. In this study, we aimed to explore the regulatory mechanisms of HYTB on the proliferation and differentiation of T lymphocytes, and the inhibitory effect of HYTB on inflammatory proliferation of FLSs. The RCS-364 (Rat FLSs) cells were cocultured with rat splenic lymphocytes that were induced by interleukin-1*β* in Transwell chambers. After freeze-dried HYTB powder treatment, the percentage of T-cell subset and apoptosis rates of FLSs were measured using flow cytometry. Furthermore, protein expression of key molecules of NF-*κ*B and JAK/STAT signaling pathways was quantified using Western blot. The granulocyte-macrophage colony-stimulating factor (GM-CSF) was measured using enzyme-linked immunosorbent assay. The results showed that HYTB could inhibit the inflammatory proliferation of FLSs through inducing cell apoptosis. Additionally, HYTB treatment could intervene in the proliferation and differentiation of T lymphocytes and regulate protein expression of key molecules in NF-*κ*B and JAK/STAT cell signaling pathways. Moreover, it could inhibit FLS activation by suppressing GM-CSF production by T cells and FLSs. Therefore, the HYTB formula should be used as a traditional medicine against RA in modern complementary and alternative therapies.

## 1. Introduction

Rheumatoid arthritis (RA) is a chronic inflammatory disease that affects 0.5%–1.0% of adults worldwide. RA joint inflammation is associated with immune cell infiltration, synovial inflammatory hyperplasia, and excessive proinflammatory mediator production, eventually resulting in articular cartilage injury [1, 2]. Although the detailed etiology and pathogenesis of RA remain uncertain, recent studies have demonstrated that inflammatory proliferation and activation of fibroblast-like synoviocytes (FLSs) and abnormal T-cell subset differentiation play critical roles in RA pathogenesis [3, 4]. T-cell activation also contributes to RA pathogenesis. Abnormal balance of T helper cells, Th1/Th2, and Treg/Th17 induces the inflammatory immune response and FLS activation. FLSs produce proinflammatory cytokines and chemokines, recruit effective T cells to synovial tissue in the joint, and initiate autoimmune arthritis. The proinflammatory cytokine secretion, including granulocyte-macrophage colony-stimulating factor (GM-CSF), initiates and augments autoimmune arthritis [5–8]. Effective Th cells and activating FLSs participate in autoimmune responses and aggravate bone and cartilage degradation [9, 10].

The nuclear factor-*κ*B (NF-*κ*B) signaling pathway is related to several pathophysiological changes including inflammation, cell survival, proliferation, and differentiation. Activation of this pathway regulates proinflammatory cytokine production and accelerates the RA pathological progression. Moreover, inflammatory cytokines activate the Janus kinase/signal transducers and activators of transcription (JAK/STAT) signaling pathway (also known as IL-6 signal pathway) and elevate the matrix metalloproteinase gene expression, resulting in apoptosis resistance in FLSs [11]. The proinflammatory cytokines activate Th17 and FLSs to secrete GM-CSF. GM-CSF stimulates monocytes, dendritic cells (DCs), and Th17 cells and augments innate and adaptive immune cell activation and the synovitis in joints [10].

Huayu Tongbi Fang (HYTB), a Chinese herbal formula, is considered to improve the RA pathological microenvironment by activating blood circulation, dissipating blood stasis, and dredging meridians and collaterals. Its curative effect and drug safety have been established through long clinical practice in RA treatment. Despite the good curative effect of HYTB in RA treatment [12], the mechanisms for inhibiting abnormal T-cell differentiation and inflammatory proliferation of FLSs are not clear. In this study, RSC-364 cells (Rat FLSs cell line) [4, 13] cocultured with rat lymphocytes that were induced by IL-1*β* in Transwell chambers were used as cell models. Following freeze-dried HYTB powder treatment, the rates of T-cell subsets and FLSs apoptosis were measured. The expression of key molecules in NF-*κ*B and JAK/STAT signaling pathways and GM-CSF level were also studied. We wanted to explore the possible mechanisms by which HYTB regulates T-cell proliferation and differentiation, thus inhibiting the FLSs activation and inflammatory proliferation.

## 2. Materials and Methods

### 2.1. Preparation of the Freeze-Dried HYTB Powder

The modified HYTB formula was composed of six medicinal herbs. The constitution ratio of the six herbs was composed of *Ligusticum chuanxiong* Hort [4], *Dioscorea nipponica Makino* [4], *Radix Aconiti Lateralis Preparata* [2], *Astragalus membranaceus* [4], *Radix Paeoniae Alba* [3], and *Corydalis yanhusuo* [3]. The herbal mixture was soaked in deionized water for 20 min and extracted with boiling water at 10 : 1 (v/w) for 2.5 h. The extracted liquid was collected, and the herb residue was then added with deionized water and boiled for another 1 h. The extracted liquid was collected and combined with the first one. After the water extract was concentrated by heating the mixture at 75°C for 2.5 h. The herbal extract was filtered using a standard test sieve of 150 *μ*m, freeze-dried, and maintained in desiccators at 4°C until use.

### 2.2. High-Performance Liquid Chromatography-Electrospray Ionization/Mass Spectrometer (HPLC-ESI/MS^n^) Analysis

The constituents of the freeze-dried HYTB powder were measured using HPLC-ESI/MS^n^. The specific measuring procedures were based on our previously described method [14]. The PeakView™ 2.2 software was used to analyze the data including retention time, accurate mass, and MS/MS spectrum comparison.

### 2.3. Cell Viability Assay

Cell viability assay was performed using the cell counting kit-8 (CCK8) method. In brief, RSC-364 cells were seeded into a 96-well plate at a density of 4,000 cells/well for 24 h at 37°C and 5% CO_2_ in a cell incubator. Following treatment with various HYTB concentrations (0, 10, 50, 100, 250, 500, 750, and 1000 *µ*g/mL, six repetitions) for 24 h at 37°C, cells were added to wells with 10 *µ*L CCK8 (Beyotime, Nanjing, China) per well and incubated for 4 h. Subsequently, optical density (OD) was measured at 450 nm using a Varioskan Flash microplate reader (Thermo Fisher Scientific, Inc.). According to the experimental results, we used the 100 and 250 *µ*g/mL concentrations as cell stimulation concentrations in this study (Figure S1).

### 2.4. Preparation of Rat Lymphocytes

Sprague Dawley rats (weighing 180–220 g) were obtained from the Beijing Vital River Laboratory Animal Technology Co. Ltd, Beijing, China (certificate number SCXK: 2012-0001). This study was approved by the Institutional Ethics Committee of the Beijing University of Chinese Medicine (No. 17-02, Beijing, China), and all animal protocols complied with the National Institutes of Health Guide for the Care and Use of Laboratory Animals (revised 2011). After intraperitoneal injection (50 mg/kg) of pentobarbital sodium anesthesia, the spleen was isolated under sterile conditions. The rats were sacrificed by intravenous pentobarbital sodium anesthesia (150 mg/kg) administration, and the death of rats after cardiac arrest was confirmed for 30 min. Single-cell suspensions from rat spleens were collected with gentle MACS Dissociator (Macs Miltenyi, Teterow, Germany). The spleens were placed in a homogenate pipe with phosphate-buffered saline (PBS). The suspension was filtered through a 70 *μ*m nylon cell strainer. After centrifugation (1000 rpm, 5 min), cells were resuspended in 5 mL of Red Blood Cell Lysis Buffer for 5 min and centrifuged at 2000 rpm for 5 minutes. The splenic lymphocytes were cultured in 2 mL RPMI-1640 medium supplemented with 3% fetal bovine serum (FBS).

### 2.5. Cell Culture and Treatment

RSC-364 cells were collected and seeded into six-well culture plates (1 × 10^6^ cells/per well). After synchronization, cells were washed three times with PBS. Transwell inserts were placed onto culture plates. The lymphocytes (1 × 10^6^ cells/per well) were then seeded into the upper chambers. The freeze-dried HYTB powder was dissolved in RPMI-1640 medium. Afterward, cells were stimulated for 12 h and 24 h in RPMI-1640 medium containing 5%. FBS, with 25 ng/mL IL-*β* alone or together with 25 ng/mL IL-1 receptor antagonist (IL-1RA) or the freeze-dried HYTB powder, at a final concentration of 100 or 250 *μ*g/mL.

### 2.6. Hematoxylin-Eosin (HE) Staining

RSC-364 cells (1.5 × 10^5^ cells/per well) were seeded into 24-well plates with preplaced coverslips. Lymphocytes (1.5 × 10^6^ cells/per well) were seeded into Transwell upper chambers in separate wells. After 12 or 24 h of treatment, RSC-364 cells were fixed with 95% alcohol for 15 min. Next, cells were stained with hematoxylin and eosin. The stained cells were observed and photographed using a light microscope (DM RAS2, Leica, Solms, Germany).

### 2.7. Flow Cytometry Analysis

RSC-364 cells were stained using an Annexin V/propidium iodide (PI) apoptosis detection kit (BD Biosciences, MA, USA). After 12 or 24 h of treatment, cells were harvested, washed three times with PBS, and incubated with 5 *μ*L Annexin V-FITC and 10 *μ*L PI for 20 min at room temperature. To measure the Th1, Th2, or Th17 cell percentages, lymphocytes were harvested and stimulated with phorbol 12-myristate 13-acetate (PMA) (50 ng/mL) and ionomycin (Ion) (1 *μ*g/mL) (Sigma, San Francisco, CA, USA) in the presence of GolgiPlug (BD Bioscience) for 5 h at 37°C and 5% CO_2_ in a cell incubator. After being surface-labeled with anti-rat CD4 PE-Cyanine5 antibody (eBioscience), lymphocytes were blocked, fixed, permeabilized using Fixation/Permeabilization kit (BD Bioscience), and stained with anti-rat IFN*γ* PE, IL-4 PE-Cyanine7, or IL-17A FITC antibodies (BD Bioscience). The stained apoptotic cells and Th cells were measured using a FACS Calibur cytometer, and data were analyzed using CellQuest software (Beckman Coulter, Brea, CA, USA).

### 2.8. Enzyme-Linked Immunosorbent Assay (ELISA)

After 12 or 24 h of treatment, the supernatant of the culture solution was collected. The GM-CSF concentrations were measured using ELISA kit (eBioscience), according to the instructions provided by the manufacturer. The optical density of each sample was measured with a plate reader at 450 nm. Subsequently, the GM-CSF levels were quantified using standard curves and shown as the number of picograms per milliliter.

### 2.9. Western Blot Analysis

After 12 or 24 h of treatment, RSC-364 cells and lymphocytes were harvested. Cells were lysed using RIPA Lysis Buffer containing protease inhibitor cocktail (Sigma, St. Louis, MO, USA). Protein samples (2 mg/ml) were separated by 10% sodium dodecyl sulfate polyacrylamide gel electrophoresis (SDS-PAGE) and electrotransferred onto the polyvinylidene difluoride membranes (Sigma, St. Louis, MO, USA). The membranes were blocked with 5% skim milk and incubated overnight with the following primary antibodies at 4°C: T-bet, GATA-3, ROR*γ*t, TRAF2, IKK*α*/*β*, phospho-IKK*α*/*β*, and NF-*κ*B p50 for lymphocytes; TRAF2, MyD88, IKK*α*/*β*, phospho-IKK*α*/*β*, NF-*κ*B p50, JAK1, STAT3, and phospho-STAT3 for RSC-364 cells (1 : 1000, Cell Signaling Technology, MA, USA). The membranes were then incubated with horseradish peroxidase- (HRP-) conjugated IgG secondary antibody (1 : 3000, Abcam, Cambridge, MA, USA) at room temperature for 2 h. All immunoreactive proteins were visualized using SuperSignal West Pico Chemiluminescent Substrate (Thermo Scientific, Rockford, IL, USA). Three replicates of each experiment were performed. The densitometry values were normalized to GAPDH and quantified using Image-Pro Plus version 4.0 (Media Cybernetics Inc., Rockville, MD, USA).

### 2.10. Statistical Analysis

All data are presented as mean ± standard deviation (SD). The SPSS version 13.0 was used for statistical analyses. A *P* < 0.05 was considered statistically significant.

## 3. Results

### 3.1. Identification of Chemical Constituents in HYTB by HPLC-ESI/MS^n^

**Figure 1:**
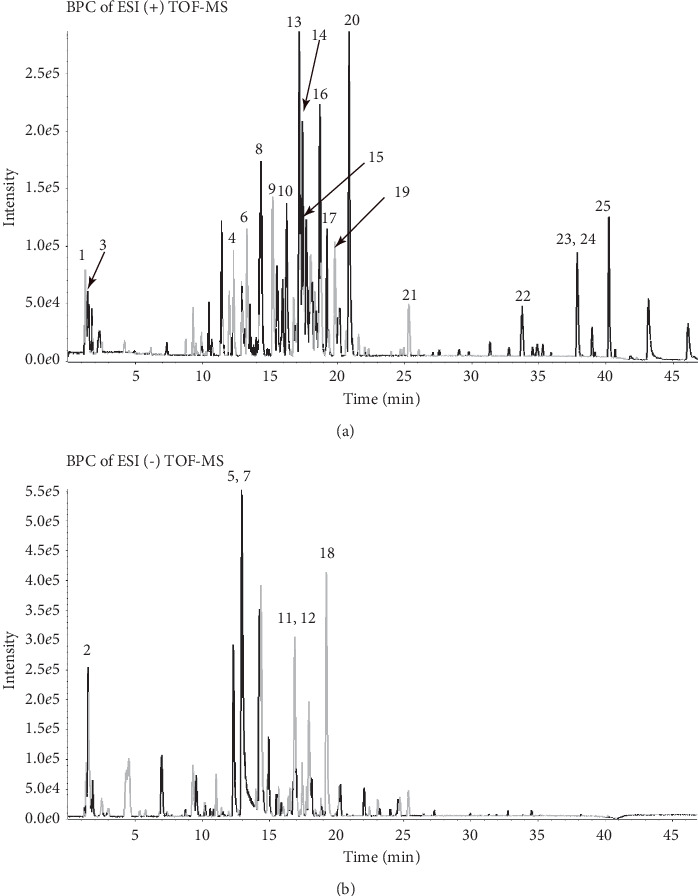
HPLC-ESI/MS^n^ total ion chromatograms of HYTB processed by different methods. (a) Negative base peak of MS spectrum. (b) Positive base peak of MS spectrum.

Representative liquid chromatography-mass spectrometry chromatograms are shown in Figure 1. Negative (Figure 1(a)) and positive (Figure 1(b)) modes were operated in the HPLC-ESI/MS^n^ experiment. Twenty-five constituents were identified by comparing the retention time with the IDA method. The identified compounds are shown in Table 1.

### 3.2. HYTB Regulated the Differentiation of Th and Treg Cells of Lymphocytes in the Coculture System Induced by IL-1*β*

**Figure 2:**
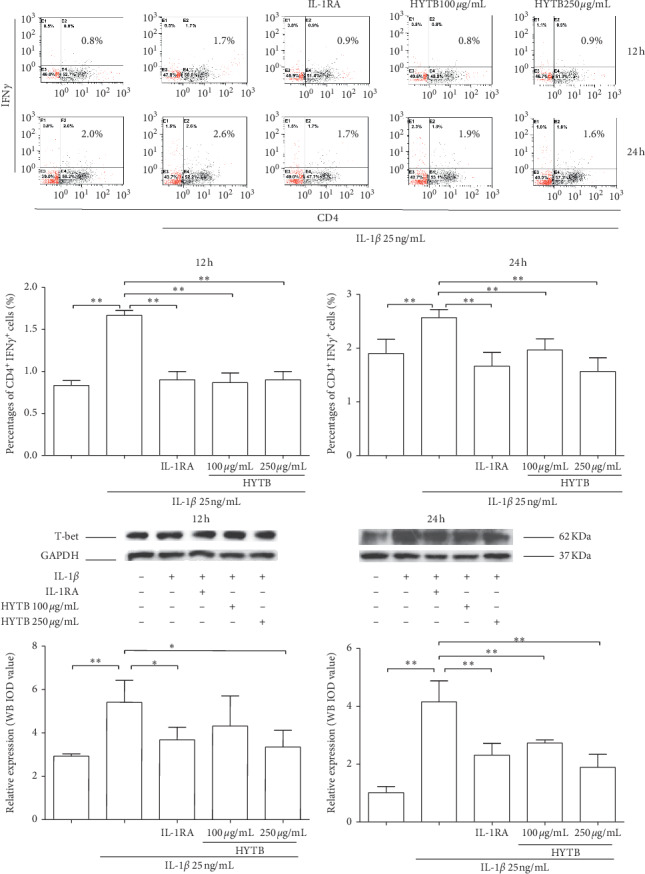
Percentages of CD4^+^IFN*γ*^+^ cells and protein expression of T-bet in lymphocytes after treatment. (a) Flow cytometry histogram. The results are presented in bar charts. (b) T-bet was detected in lymphocytes by Western blot analysis. The quantified results are presented in bar charts. GAPDH is used as an internal control. Data are presented as the mean ± SD (*n* = 3). ^*∗*^*P* < 0.05 and ^*∗∗*^*P* < 0.01.

**Figure 3:**
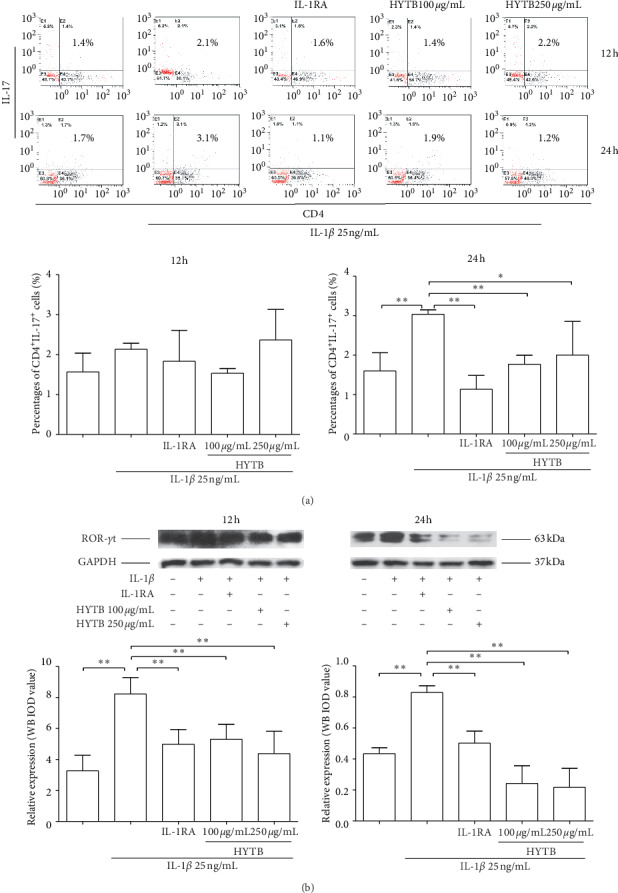
Percentages of CD4^+^IL-17^+^ cells and protein expression of ROR*γ*t in lymphocytes after treatment. (a) Flow cytometry histogram. The results are presented in bar charts. (b) ROR*γ*t was detected in lymphocytes by Western blot analysis. The quantified results are presented in bar charts. GAPDH is used as an internal control. Data are presented as the mean ± SD (*n* = 3). ^*∗*^*P* < 0.05 and ^*∗∗*^*P* < 0.01.

**Figure 4:**
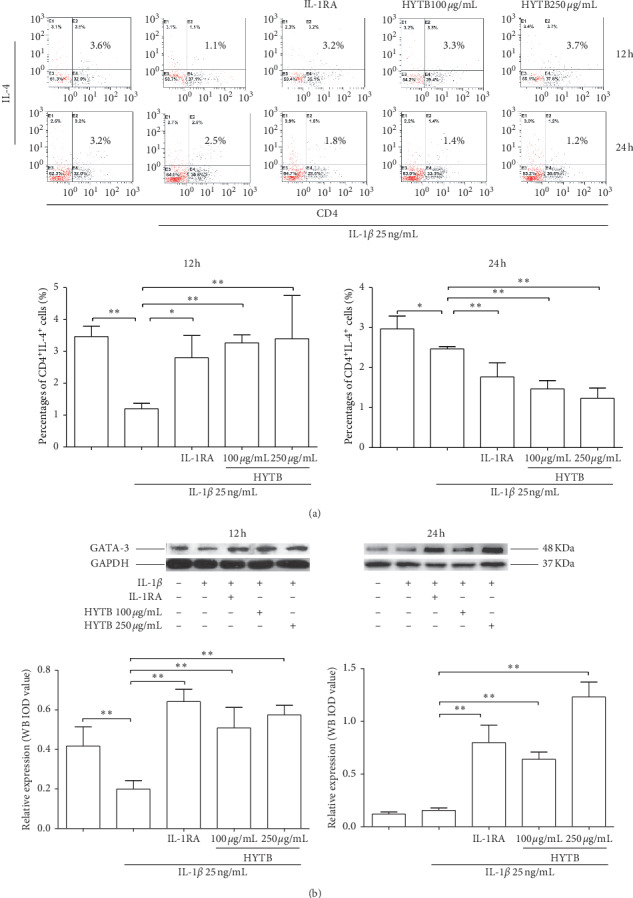
Percentages of CD4^+^IL-4^+^ cells and protein expression of GATA-3 in lymphocytes after treatment. (a) Flow cytometry histogram. The results are presented in bar charts. (b) GATA-3 was detected by Western blot analysis. The quantified results are presented in bar charts. GAPDH is used as an internal control. Data are presented as the mean ± SD (*n* = 3). ^*∗∗*^*P* < 0.01.

**Figure 5:**
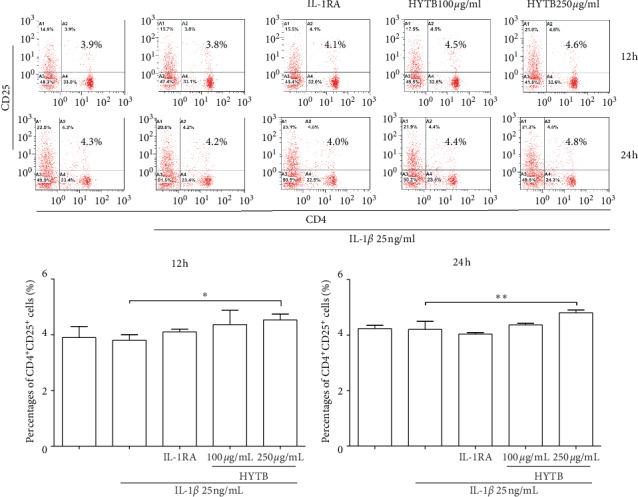
Percentages of CD4^+^CD25^+^ cells after treatment. Flow cytometry histogram. The results are presented in bar charts. Data are presented as the mean ± SD (*n* = 3). ^*∗*^*P* < 0.05 and ^*∗∗*^*P* < 0.01.

The percentages of Th1, Th2, Th17, and Treg cells in lymphocytes were measured by flow cytometry. The Th1 (IFN^+^CD4^+^, Figure 2(a)) and Th17 (IL-17^+^CD4^+^, Figure 3(a)) cell percentages were significantly increased compared with those of the model group after 12 and 24 h of IL-1*β* induction. After 12 or 24 h of HYTB treatment, the abnormal differentiation of Th1 and Th17 cells was suppressed. However, the percentages of Th2 (IL-4^+^CD4^+^, Figure 4(a)) cells were significantly decreased by IL-1*β*. After 12 h of HYTB treatment, the number of Th2 cells was significantly increased. The percentages of Treg (CD4^+^CD25^+^, Figure 5) cells in the HYTB group (250 *μ*g/mL) were significantly increased compared to those induced by IL-1*β* alone at these two-time nodes of treatment. These results indicated that HYTB treatment could interfere with the proliferation and differentiation of Th and Treg cells induced by IL-1*β*, and the regulatory effects were time- and dose-dependent.

To explore HYTB treatment mechanisms on intervening in the proliferation and differentiation of Th cells, the protein expression of specific transcription factors in Th cells was measured using Western blot analysis. As shown in Figures 2(b), 3(b), and 4(b), the T-bet protein levels (the specific transcription factor of Th1) and ROR*γ*t (that of Th17) were significantly decreased, and GATA-3 (that of Th2) was remarkably increased in lymphocytes after HYTB treatment at two-time nodes. The regulatory effects of HYTB treatment, especially of 250 *μ*g/mL HYTB treatment groups, were equal or superior to those of IL-1RA groups.

### 3.3. HYTB Inhibited the Activation of the NF-*κ*B Signaling Pathway in Lymphocytes Induced by IL-1*β*

**Figure 6:**
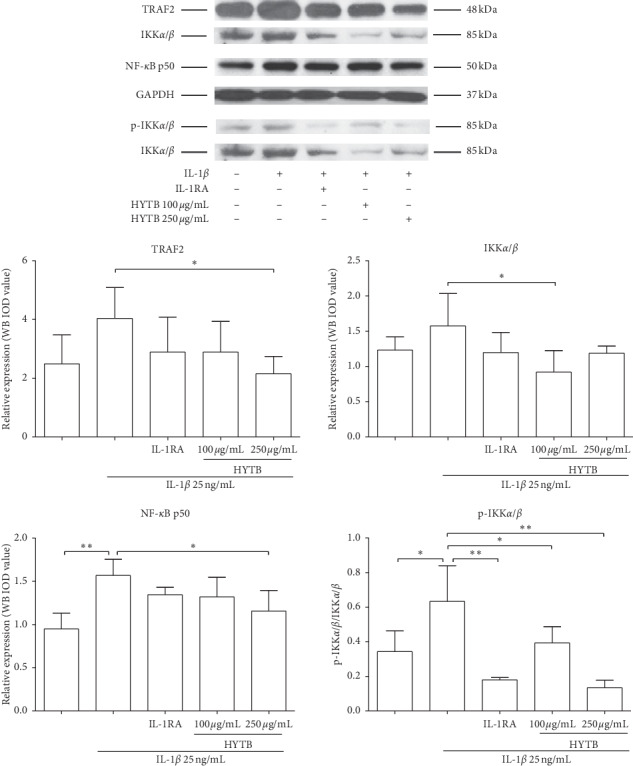
Effect of HYTB on inhibiting the NF-*κ*B signaling pathway activation in lymphocytes after 12 h of treatment. The expression levels of TRAF2, IKK*α*/*β*, p-KK*α*/*β*, and NF-*κ*B p50 in lymphocytes were measured using the Western blot analysis. The quantified results are presented in bar charts. GAPDH is used as an internal control. Data are presented as the mean ± SD (*n* = 3). ^*∗*^*P* < 0.05 and ^*∗∗*^*P* < 0.01.

**Figure 7:**
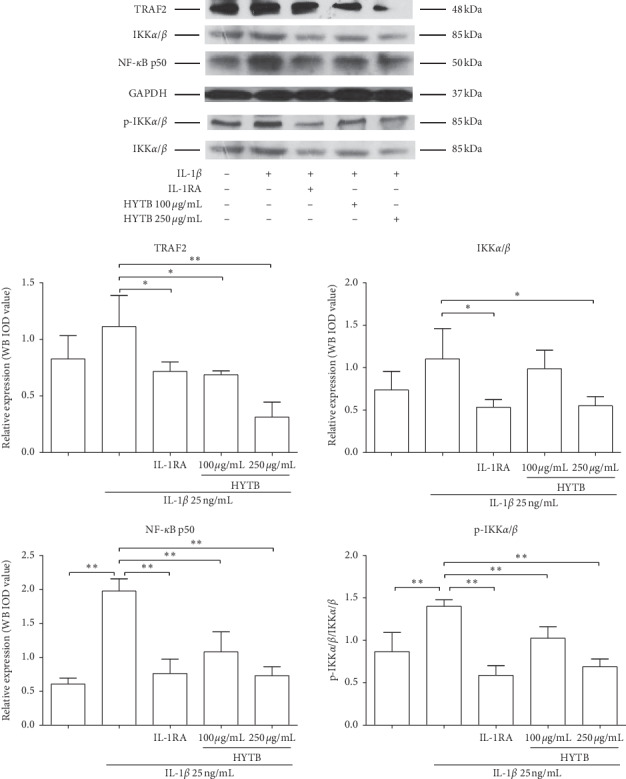
Effect of HYTB on inhibiting the NF-*κ*B signaling pathway activation in lymphocytes after 24 h of treatment. The expression levels of TRAF2, IKK*α*/*β*, p-KK*α*/*β*, and NF-*κ*B p50 in lymphocytes were measured using Western blot analysis. The quantified results are presented in bar charts. GAPDH is used as an internal control. Data are presented as the mean ± SD (*n* = 3). ^*∗∗*^*P* < 0.01.

IL-1*β* can activate the NF-*κ*B signaling pathway and contribute to the inflammatory immune response [15]. We measured the protein levels of key molecules in the NF-*κ*B signaling pathway of lymphocytes after HYTB treatment. As shown in Figures 6 and 7, the protein levels of TRAF2, IKK*α*/*β*, and NF-*κ*B p50 were significantly downregulated after 12 and 24 h of HYTB treatment. Phospho-IKK*α*/*β* also significantly decreased. The HYTB inhibitory effects (250 *μ*g/mL) of the treatment groups were better than those of the other groups after 24 h of treatment.

### 3.4. HYTB Suppressed the Inflammatory Proliferation of FLSs by Inducing Cell Apoptosis

**Figure 8:**
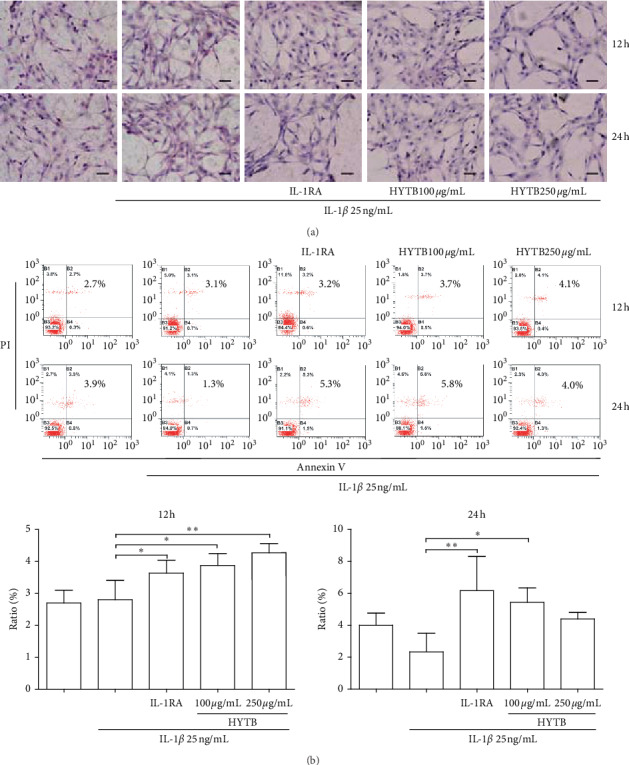
HYTB treatment attenuated the FLSs inflammatory proliferation induced by IL-1*β*. (a) Morphology analysis (hematoxylin and eosin-stained section) of RSC-364 cells (200x). (b) Proapoptotic effect of HYTB on IL-1*β*-stimulated RSC-364 cells. Flow cytometric analysis demonstrating the effect of HYTB apoptosis induction. The results are presented in bar charts. Data are presented as the mean ± SD (*n* = 3). ^*∗*^*P* < 0.05 and ^*∗∗*^*P* < 0.01.

The HE staining analysis showed that RSC-364 cells appeared regular with large and spindle-shaped nuclei in normal group, and cells induced by IL-1*β* exhibited irregular spindle shape and various degrees of damage in the cytoplasm. The inflammatory proliferation of RSC-364 cells also appeared. HYTB treatment significantly inhibited FLSs proliferation and repaired the cell damage (Figure 8(a)).

In addition, we also measured apoptotic cell percentage in RSC-364 cells after treatment. As shown in Figure 8(b), the apoptotic cell percentage in the HYTB treatment groups significantly increased compared with that induced by IL-1*β* alone after 12 h of treatment. The inducing effect of HYTB treatment was superior to that of IL-1RA.

### 3.5. HYTB Treatment Inhibited GM-CSF Production in the Coculture System of Lymphocytes and FLSs Induced by IL-1*β*

**Figure 9:**
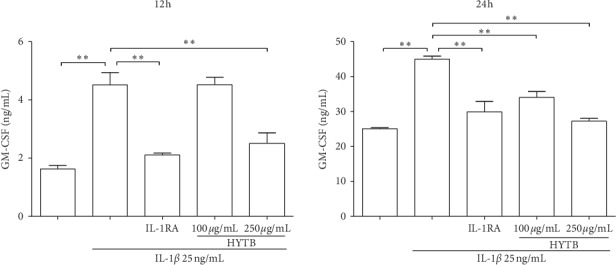
The GM-CSF levels in the coculture supernatant were quantified by ELISA. Data are presented as the mean ± SD (*n* = 3). ^*∗∗*^*P* < 0.01.

IL-1*β* signaling drives Th17 cells and FLSs to produce GM-CSF, active DCs, and macrophages, contributing to perpetuate autoimmune inflammation [16, 17]. We measured the GM-CSF levels in the supernatant of the coculture solution after treatment by ELISA. As shown in Figure 9, the GM-CSF levels in a coculture system of lymphocytes and FLSs significantly increased after IL-1*β* stimulation. HYTB treatment downregulated the GM-CSF secretion. The HYTB treatment inhibitory effect at 250 *μ*g/mL concentration was equal or superior to that of IL-RA.

### 3.6. Inhibitory Effects of HYTB on the Activation of NF-*κ*B and JAK/STAT Signaling Pathways of RSC-364 Cells in the Transwell System

**Figure 10:**
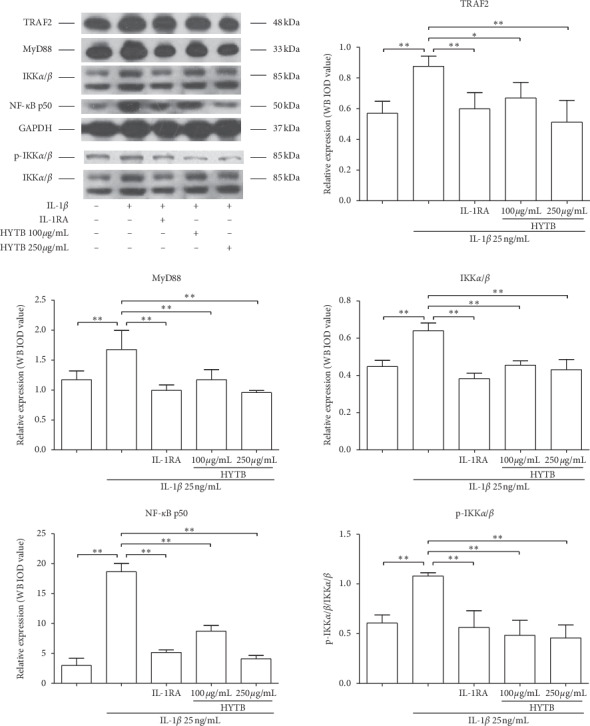
The key molecules protein levels of the NF-*κ*B signaling pathway in RSC-364 cells after 12 h of treatment. TRAF2, MyD88, IKK*α*/*β*, p-IKK*α*/*β*, and NF-*κ*B p50 were quantified using Western blot analysis. The quantified results are presented in bar charts. GAPDH is used as an internal control. Data are presented as the mean ± SD (*n* = 3). ^*∗*^*P* < 0.05 and ^*∗∗*^*P* < 0.01.

**Figure 11:**
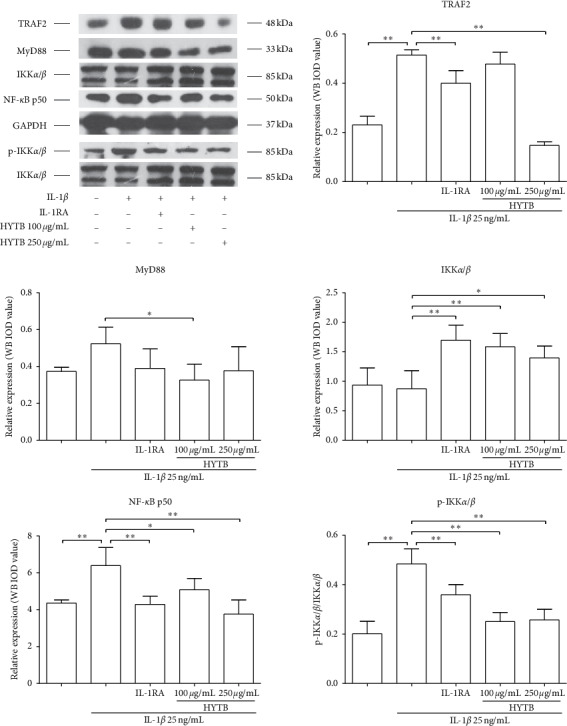
The key molecules protein levels of the NF-*κ*B signaling pathway in RSC-364 cells after 24 h of treatment. TRAF2, MyD88, IKK*α*/*β*, p-IKK*α*/*β*, and NF-*κ*B p50 were detected using Western blot analysis. The quantified results are presented in bar charts. GAPDH was used as an internal control. Data are presented as the mean ± SD (*n* = 3). ^*∗*^*P* < 0.05 and ^*∗∗*^*P* < 0.01.

**Figure 12:**
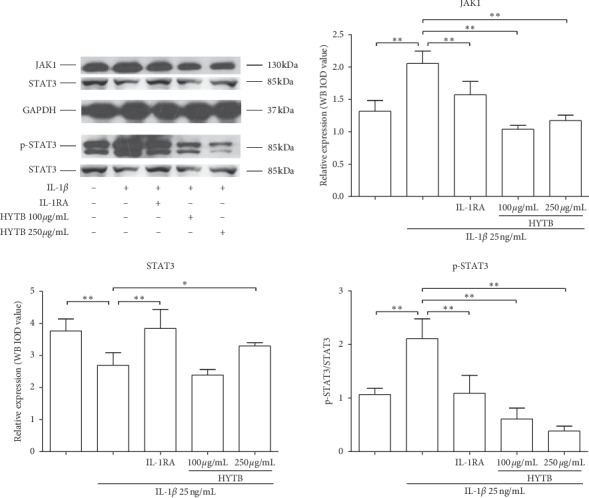
The key molecules protein levels of JAK/STAT signal pathway in RSC-364 cells after 12 h of treatment. JAK1, STAT3, and p-STAT3 were detected using Western blot analysis. The quantified results are presented in bar charts. GAPDH is used as an internal control. Data are presented as the mean ± SD (*n* = 3). ^*∗*^*P* < 0.05 and ^*∗∗*^*P* < 0.01.

**Figure 13:**
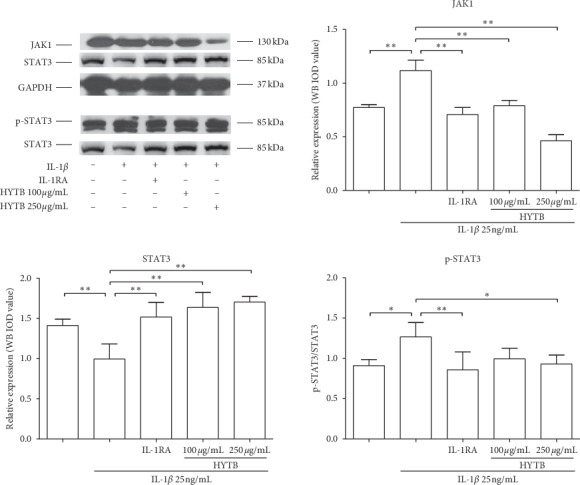
The key molecules protein levels of JAK/STAT signal pathway in RSC-364 cells after 24 h of treatment. JAK1, STAT3, and p-STAT3 were detected using Western blot analysis. The quantified results are presented in bar charts. GAPDH is used as an internal control. Data are presented as the mean ± SD (*n* = 3). ^*∗*^*P* < 0.05 and ^*∗∗*^*P* < 0.01.

Activation of NF-*κ*B and JAK/STAT signaling pathways plays key roles in the RA pathogenesis [18]. For these reasons, we measured the protein levels of key molecules in NF-*κ*B and JAK/STAT signaling pathways in RSC-364 cells after treatment. As shown in Figures 10 and 11, the protein expression of MyD88, TRAF2, IKK*α*/*β*, and NF-*κ*B p50 in RSC-364 cells of HYTB treatment groups was significantly lower than those induced by IL-1*β* alone at two time nodes. Furthermore, IKK*α*/*β* protein phosphorylation was inhibited by HYTB treatment. The phospho-STAT3 and JAK1 protein levels in RSC-364 cells also significantly decreased after HYTB treatment (Figures 12 and 13). The inhibitory effects of 250 *μ*g/mL HYTB were better than those of IL-1RA groups.

## 4. Discussion

In traditional Chinese medicine, multiple agents contained in one formula will synergistically work as medical treatment. Twenty-five chemical constituents of HYTB formula were identified by HPLC-ESI/MS^n^ analysis. Pharmacological research demonstrated that paeoniflorin can inhibit lipopolysaccharide-induced production of tumor necrosis factor-*α* (TNF-*α*) and IL-1*β*, thereby increasing IL-10 production [19]. Coptisine can inhibit IL-1*β*-induced inflammatory response by suppressing NF-*κ*B signaling pathway [20]. Moreover, isoschaftoside and jatrorrhizine have been shown to inhibit proliferation, migration, and production of inflammatory mediators [21, 22]. Epiberberine, coptisine, and p-coumaric acid have antibacterial, anti-inflammatory, and antioxidative effects [23–26]. Pharmacological studies have also shown that calycosin-7-O-glucoside could reduce the expression of platelet-derived growth factor, fibroblast growth factor, and toll-like receptor [27]. Tanshinone IIA could upregulate lncRNA GAS5 and block cell cycle in the G2/M phase, inducing FLSs apoptosis [28, 29]. Therefore, we suggest that these HYTB constituents could be contributed by interacting with multiple targets and exerting synergistic therapeutic effects on regulating proliferation and differentiation of Th and Treg cells, thereby inhibiting inflammatory FLSs proliferation by inducing apoptosis, and attenuating proinflammatory cytokine production for the suppression and resolution of inflammation in RA.

T-cell infiltration and inflammatory cytokine imbalance in the joint lumen could induce an abnormal anti-immune response and initiate inflammatory proliferation of synovial tissue and the autoimmune arthritis, resulting in articular cartilage injury [30]. The imbalances in the differentiation and function of Th1 and Th2/Th17 cells influence RA pathogenesis [3, 31, 32]. Naïve CD4^+^ T cells differentiate into either type of Th cell subset depending on the stimulation of specific transcription factors. Th1 cell differentiation depends on expression of transcription factor T-bet through the IL-12/STAT-4 signaling pathway and the interferon-*γ* (IFN-*γ*)/STAT-1 signaling pathway [33]. GATA-3 induces Th2 cell differentiation by the IL-4/STAT-6 pathway [34]. Th17 cell differentiation induces the expression of the transcription factor ROR*γ*t by TGF*β* and IL-6/STAT3 pathways [35].

Th1 cells play a predominant role in the RA pathology. Synovium-infiltrating Th1 cells secrete IFN-*γ* and activate macrophages and TNF production [36]. However, several studies have demonstrated that Th1 phenotype does not explain all mechanisms involved in RA. IL-17-producing Th17 cells were found in peripheral blood monocytes in a large proportion of RA patients. The Th17 cell proportion is related to disease activity during RA progression [36]. IL-17 secreted by Th17 cells activates osteoblasts and FLSs and induces osteoclastogenesis [37]. IL-17 also induces production of TNF and IL-6 from FLSs and macrophages and augments recruitment of inflammatory cells into the synovial tissue. CD4^+^ T cells overexpressing ROR*γ*t induce the CCR6 production and promote the CD4^+^ T-cell migration into inflamed joints [38]. Th2 cells coordinating with IL-4 and IL-10 suppress Th1 cell differentiation. In addition, IL-4R controls IL-17 production. GATA-3 overexpression reduces Th17 cell differentiation [39]. Treg cells have regulatory roles in the development of autoimmune arthritis. Depletion of CD25^+^ T cells augments the severity of collagen-induced arthritis in a murine model. CD4^+^CD25^+^ Treg cell transplantation suppresses the progression of arthritis [40]. In this study, our results indicated that HYTB treatment has a regulatory effect on Th cells differentiation. HYTB could downregulate Th1 and Th17 cell proportions and induce Th2 and Treg cell differentiation after IL-1*β* stimulation. One possible mechanism is interfering with protein expression of Th specific transcription factors.

Synovial hyperplasia and infiltration of immune cells lead to excessive expansion and destruction of articular cartilage in RA [41]. Activated FLSs have resistance to cell apoptosis. Small molecule inflammatory mediators and proteolytic enzymes produced by FLSs can degrade the extracellular matrix in RA [42]. Our results showed that HYTB treatment could inhibit FLSs inflammatory proliferation induced by IL-1*β* by promoting apoptosis.

GM-CSF, a key proinflammatory cytokine, activates dendritic cells and macrophages. Th17 cells induced by IL-1 and IL-23 signaling secrete GM-CSF and initiate autoimmune inflammation [43]. FLSs are important effector cells that produce large amounts of inflammatory cytokines, contributing to cartilage and bone degradation in RA. IL-17 combined with IL-1 increases GM-CSF production by FLSs. Loss of GM-CSF production by Th17 cells and FLSs could inhibit progression of autoimmune arthritis [44]. HYTB treatment decreased GM-CSF production in the coculture system of lymphocytes and FLSs induced by IL-1*β*.

The NF-*κ*B signaling pathway plays a key role in the pathogenesis of RA. NF-*κ*B signaling pathway activation stimulates proinflammatory cytokines expression, leading to the inflammatory response of RA [45]. It can also induce expression of antiapoptotic cytokines, inhibit apoptosis of FLSs, and contribute to the synoviocyte hyperplasia stimulated by proinflammatory cytokines [46]. The JAK/STAT3 signaling pathway also plays a key role in the RA pathological progression. The cell cycle and apoptosis of FLSs in RA are regulated by activation of JAK/STAT3 signaling pathway [47]. The results of present study showed that HYTB treatment could intervene in the protein expression of key molecules of NF-*κ*B and JAK/STAT3 signaling pathways, inhibit activation of these pathways, induce FLSs apoptosis, and contribute toward inhibiting inflammatory proliferation of FLSs induced by proinflammatory cytokines.

Our study demonstrated that the HYTB formula could regulate proliferation and differentiation of Th cells, suppress NF-*κ*B and JAK/STAT signaling pathways activation, induce apoptosis in FLSs, and decrease GM-CSF production, resulting in the suppression of inflammatory proliferation of FLSs stimulated by proinflammatory cytokines. HYTB can restore homeostasis in the tissue microenvironment of RA. These in vitro experimental results and preliminary clinical observation results provide supporting supplements for HYTB formula in the treatment of autoimmune arthritis, including RA. However, *in vivo* experiments and clinical trials are necessary to confirm the anti-inflammatory activities of HYTB. This is also a key point in our follow-up research work. Taken together, this Chinese medical formula, HYTB, should be used as a complementary or alternative traditional medicine for RA treatment.

## Figures and Tables

**Table 1:** Chemical components identified from HYTB by HPLC-ESI/MS^n^.

Peak	Ionization mode	TR (min)	Formula	Identification	Peak area	Area (%)
1	+	1.46	C_5_H_9_NO_2_	Proline	1642000	1.8
2	−	1.73	C_7_H_10_O_5_	Shikimic acid	690900	1.3
3	+	1.79	C_5_H_11_NO_2_	L-Valine	76360	0.1
4	+	12.3	C_23_H_28_O_11_	Albiflorin	3810000	4.2
5	−	12.84	C_26_H_28_O_14_	Isoschaftoside	177500	0.3
6	+	12.93	C_23_H_28_O_11_	Paeoniflorin	4280000	4.7
7	−	13.27	C_9_H_8_O_3_	p-Coumaric acid	140300	0.3
8	+	14.21	C_22_H_22_O_10_	Calycosin-7-O-glucoside	2572000	2.8
9	+	15.21	C_20_H_23_NO_4_	Tetrahydrojatrorrhizine	7699000	8.4
10	+	16.24	C_20_H_19_NO_5_	Protopine	7553000	8.3
11	−	16.5	C_7_H_6_O_3_	4-Hydroxybenzoic acid	813700	1.5
12	−	16.88	C_18_H_16_O_8_	Rosmarinic acid	13000000	23.7
13	+	17.52	C_20_H_20_NO_4_	Jatrorrhizine	7553000	8.3
14	+	17.6	C_20_H_18_NO_4_	Epiberberine	5307000	5.8
15	+	17.71	C_19_H_14_NO_4_	Coptisine	11150000	12.2
16	+	18	C_21_H_22_O_9_	Liquiritin	906500	1.0
17	+	18.72	C_22_H_27_NO_4_	Corydaline	11150000	12.2
18	−	19.25	C_26_H_22_O_10_	Salvianolic acid A	2482000	4.5
19	+	19.95	C_23_H_28_O_10_	Isomucronulatol-7-O-glucoside	636600	1.2
20	+	20.88	C_22_H_24_NO_4_	Dehydrocorydaline	3605000	4.0
21	+	25.34	C_16_H_12_O_4_	Formononetin	2911000	3.2
22	+	33.75	C_18_H_14_O_3_	Dihydrotanshinone I	2155000	2.4
23	+	37.86	C_19_H_20_O_3_	Cryptotanshinone	5041000	5.5
24	+	37.89	C_18_H_12_O_3_	Tanshinone I	1663000	1.8
25	+	40.2	C_19_H_18_O_3_	IIA Tanshinone	5269000	5.8

## Data Availability

The data used to support the findings of this study are included within the article and the Supplementary Materials.

## Supplementary Materials

Supplementary Materials Figure S1: cell viability of RSC-364 cells treated with HYTB. The results were presented in a bar chart. Data were presented as mean ± SD, *n* = 6.
