# Supplementary material for: Chinese Herbal Formula, Huayu Tongbi Fang, Attenuates Inflammatory Proliferation of Rat Synoviocytes Induced by IL-1β by Regulating Proliferation and Differentiation of T Lymphocytes
Source: Evid Based Complement Alternat Med. 2020 May 19;2020:1706837. doi: 10.1155/2020/1706837 (PMC7256709; doi:10.1155/2020/1706837)
Supplement: Supplementary Materials — Figure S1: cell viability of RSC-364 cells treated with HYTB. The results were presented in a bar chart. Data were presented as mean ± SD, n = 6. [file 1706837.f1.pdf]

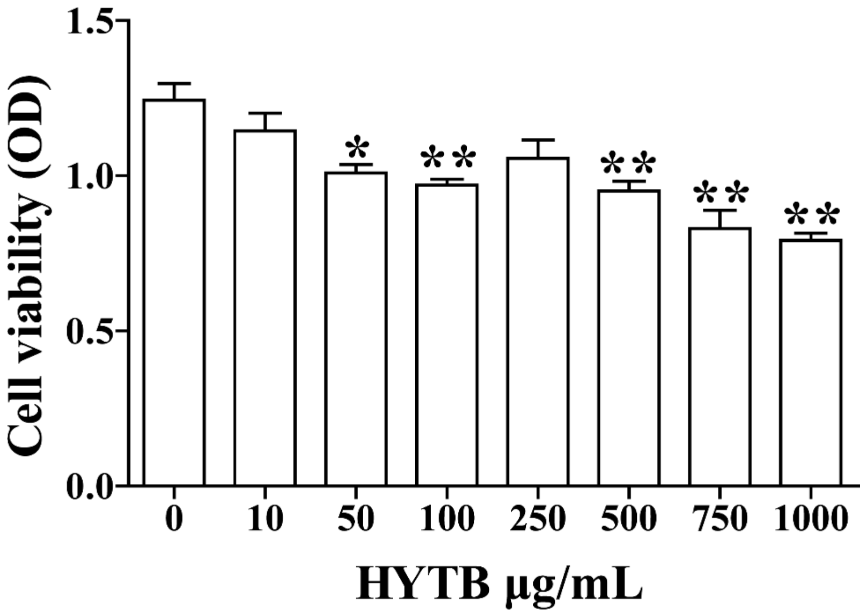

Figure S1: The cell viability of RSC-364 cells treated by HYTB. Results were presented in a bar chart. Data were presented as mean ±SD, n=6.
